# Biocatalytic C-C Bond Formation for One Carbon Resource Utilization

**DOI:** 10.3390/ijms22041890

**Published:** 2021-02-14

**Authors:** Qiaoyu Yang, Xiaoxian Guo, Yuwan Liu, Huifeng Jiang

**Affiliations:** 1 Key Laboratory of Systems Microbial Biotechnology, Tianjin Institute of Industrial Biotechnology, Chinese Academy of Sciences, Tianjin 300308, China; yangqy@tib.cas.cn (Q.Y.); guoxx@tib.cas.cn (X.G.); 2 National Technology Innovation Center of Synthetic Biology, Tianjin 300308, China; 3 University of Chinese Academy of Sciences, Beijing 100049, China

**Keywords:** C1 resource utilization, carboxylases, C-C ligases, designed pathway

## Abstract

The carbon-carbon bond formation has always been one of the most important reactions in C1 resource utilization. Compared to traditional organic synthesis methods, biocatalytic C-C bond formation offers a green and potent alternative for C1 transformation. In recent years, with the development of synthetic biology, more and more carboxylases and C-C ligases have been mined and designed for the C1 transformation in vitro and C1 assimilation in vivo. This article presents an overview of C-C bond formation in biocatalytic C1 resource utilization is first provided. Sets of newly mined and designed carboxylases and ligases capable of catalyzing C-C bond formation for the transformation of CO_2_, formaldehyde, CO, and formate are then reviewed, and their catalytic mechanisms are discussed. Finally, the current advances and the future perspectives for the development of catalysts for C1 resource utilization are provided.

## 1. Introduction

It has been estimated that more than 35% of industrial chemicals will be produced by bio-manufacturing until 2030 [1]. C1 resources, including methane, methanol, formaldehyde, formic acid, and carbon dioxide, are ideal raw materials for bio-manufacturing, due to their low cost and easy availability. In nature, six CO_2_ fixation pathways have been identified in photoautotrophic and chemoautotrophic microorganisms [2]. In addition to Calvin–Benson–Bassham (CBB) cycle, there are three major pathways that operate in methanotrophs or methylotrophic yeast for assimilation of methane or methanol, which are first oxidized to formaldehyde, and then is assimilated via the ribulose monophosphate (RuMP) cycle, serine cycle, or xylulose monophosphate pathway (XuMP) [3]. Engineering natural C1 assimilation pathways to achieve the production of biofuels or industrial chemicals has always been a research hotspot in synthetic biology [4,5,6,7,8,9,10,11,12,13,14,15,16]. However, the biotransformation of C1 resources is restricted in the industry by low production efficiency, limited product types, and high production costs.

With the development of synthetic biology, more and more C1 utilization pathways have been exploited in recent years. These pathways can be broadly divided into two categories. One refers to C1 transformation in vitro. Carboxylases and C-C ligases from naturally occurring C1 assimilation pathways are highly specialized, and thus, restricted their use for the C1 biotransformation in vitro. It has been shown that (de)carboxylases involved in the secondary metabolism are generally reversible and promiscuous [17], several C-C ligases, such as aldolases and thiamine diphosphate (ThDP)-dependent enzymes [18], can receive formaldehyde as a receptor. These newly mined (de)carboxylases and C-C ligases greatly expand the scope of C1 resource utilization in vitro. The other refers to C1 assimilation in vivo. Based on highly active carboxylases or designed C-C ligases, several artificial C1 assimilation pathways have been constructed, such as malyl-CoA-glycerate pathway (MCG) [19], crotonyl-CoA/ethylmalonyl-CoA/hydroxybutyryl-CoA cycle pathway (CETCH) [20,21], formolase pathway (FLS) [22], and synthetic acetyl-CoA pathway (SACA) [23]. Compared to the natural C1 assimilation pathways, these artificial pathways have obvious advantages in catalytic efficiency, biomass yield, and driving force, and are expected to improve the efficiency of C1 resource utilization in the future.

The overall efficiency of C1 transformation in vitro and C1 assimilation in vivo is generally determined by the biochemical properties of carboxylases and C-C ligases. A better understanding of the catalytic mechanisms of these enzymes is necessary for mining or design more efficient carboxylases and C-C ligases. Herein, we mainly summarized carboxylases, and C-C ligases for formaldehyde, CO, and formic acid. Furthermore, C1 transformation in vitro and C1 assimilation in vivo based on newly mined or designed carboxylases and C-C ligases are highlighted. Finally, a summary about the current advances and the future perspectives of the C1 resource utilization are presented.

## 2. Carboxylases for CO_2_ Biotransformation

CO_2_ is a poor electrophile and usually exists as bicarbonate in an aqueous solution. Therefore, the carboxylation reaction often requires energy (adenosine triphosphate (ATP), nicotinamide adenine dinucleotide phosphate (NADPH), or ferredoxin) or the assistance of coenzymes (metal ion, ThDP, and prenylated flavin mononucleotide (prFMN), etc.) [24]. We divide carboxylases into seven categories: (1) Only divalent metal-dependent carboxylases, (2) ATP-dependent carboxylases, (3) redox equivalents-dependent carboxylases, (4) substrate-activated carboxylases, (5) ThDP-dependent carboxylases, (6) multi-enzyme complex constructed carboxylase, (7) prFMN-dependent carboxylases. Representative carboxylases are shown in Table 1.

### 2.1. Only Divalent Metal-Dependent Carboxylases

**Figure 1:**
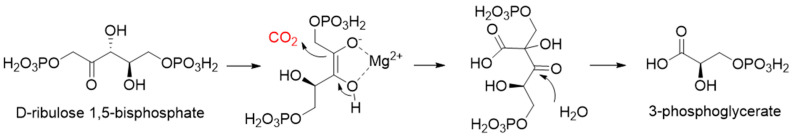
The catalytic mechanism of Rubisco [26].

The CBB cycle is the most important CO_2_ assimilation pathway on earth, and is used by most photosynthetic organisms (such as plants, algae, cyanobacteria, and most aerobic or facultative aerobic *Eubacteria*) to assimilate CO_2_ into biomass [25]. D-ribulose-1, 5-bisphosphate carboxylase/oxygenase (Rubisco, EC 4.1.1.39) is the key carboxylase, which converts CO_2_ and ribulose-1, 5-bisphosphate to 3-phosphoglycerate [26]. The catalytic process of Rubisco is that the enolate form of ribulose-1, 5-bisphosphate launches a nucleophilic attack onto CO_2_ assisted by an essential Mg^2+^, to produce a labile C6-β-ketoacid intermediate, which is hydrolytically cleaved into 3-phosphoglycerate (Figure 1). RubisCO shows two major flaws. One is the low catalytic activity, which has an average turnover number of 5 s^−1^ [27,28]. The other is a side reaction with O_2_, causing carbon loss.

Due to the high complexity of the catalytic mechanism and the unique position of RubisCO in biosynthesis, it is unlikely that this enzyme can accept non-natural substrate analogs to produce carboxylic acids. In addition to engineering cyanobacteria or microalgae to produce various biofuels and industrial chemicals, the reconstruction of the CBB cycle based on RubisCO in industrial microbial model strains (*Escherichia coli,* etc.) has made a series of progress [29,30,31]. By co-expressing Rubisco, phosphoribulokinase, and formate dehydrogenase, Shmuel Gleizer et al. engineered *E. coli* to produce all its biomass carbon from CO_2_ via the CBB cycle [30]. By adding eight heterologous genes and deleting three native genes, Thomas Gassler et al. engineered the peroxisomal methanol-assimilation pathway of *P. pastoris* into a CO_2_-fixation pathway resembling the CBB cycle [31], the resulting strain can grow continuously with CO_2_ as a sole carbon source at a μ_max_ of 0.008 h^−1^.

### 2.2. ATP-Dependent Carboxylases

**Figure 2:**
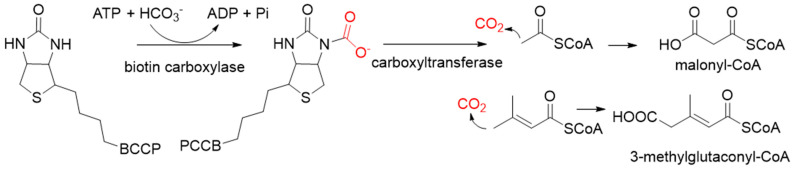
The catalytic process of biotin-dependent carboxylases [33,34,35].

Biotin-dependent carboxylases include pyruvate carboxylase (PC, EC 6.4.1.1), acetyl-CoA carboxylase (ACC, EC 6.4.1.2), propionyl-CoA carboxylase (PCC, EC 6.4.1.3), 3-methylcrotonoyl-CoA carboxylase (MCC, EC 6.4.1.4), and geranoyl-CoA carboxylase (GCC, EC 6.4.1.5). They are widely distributed in nature and can be found in archaea, bacteria, algae, fungi, plants, and animals [32]. The catalytic process of biotin-dependent carboxylases can be divided into two steps (Figure 2). First, the biotin carboxylase (BC) domain catalyzes the ATP-dependent carboxylation of the N1′ atom of the biotin cofactor, using bicarbonate as the CO_2_ donor. Second, the carboxyltransferase (CT) domain transfers the CO_2_ from carboxy-biotin to the substrates [33,34,35]. The site for carboxylation is on the α-carbon of saturated substrates (pyruvate, acetyl-CoA, and propionyl-CoA) or the γ-carbon of α, β-unsaturated substrates (3-methylcrotonyl-CoA, geranyl-CoA). Acetyl-CoA carboxylase and propionyl-CoA carboxylase are two carboxylases of 3-hydroxypropionate/malyl-CoA cycle and 3-hydroxypropionate/4-hydroxybutyrate cycle [36,37].

Acetone carboxylases (AC, EC 6.4.1.6) are soluble cytoplasmic enzymes, and can be found in many species of aerobic, anaerobic phototrophic bacteria, and even microaerobic gastric human pathogenic species *Helicobacter pylori*. They catalyze the carboxylation of acetone to form acetoacetate at the expense of ATP [38]. There are two different types of acetone carboxylases. One requires 2 ATP equivalents as an energy supply for the carboxylation reaction, while another requires 4 ATP equivalents. The main difference in catalytic mechanism lies in the processes of substrate activation. The catalytic mechanism proposed for acetone carboxylase of *Xanthobacter/Rhodobacter* is that one ATP is sequentially hydrolyzed to ADP and AMP to activate acetone and bicarbonate, respectively. While the catalytic mechanism of acetone carboxylase from *Aromatoleum* is that 2 ATP are hydrolyzed to 2 AMP to active two substrates [39]. Acetoacetate can be activated by a CoA ligase to form acetoacetyl-CoA, which is cleaved to form 2 acetyl-CoA by thiolase. Therefore, a new pathway from isopropanol and CO_2_ to acetyl-CoA can be constructed. Acetophenone carboxylase (APC, EC 6.4.1.8) catalyzes the carboxylation of acetophenone to benzoylacetate [40]. Different from the above two activation processes of acetone carboxylases, acetophenone and bicarbonate are all activated by hydrolyzing ATP to ADP.

### 2.3. Redox Equivalents-Dependent Carboxylases

**Figure 3:**
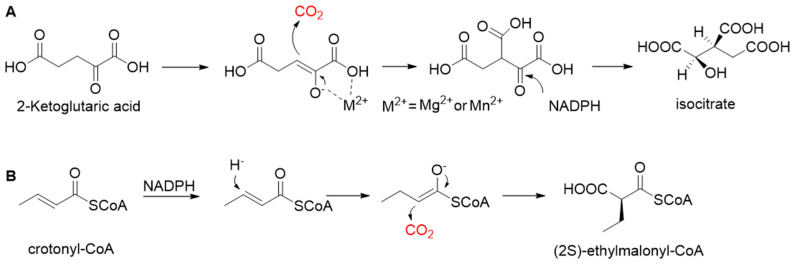
The catalytic mechanism of isocitrate dehydrogenase and crotonyl-CoA carboxylase/reductase. (**A**) The catalytic mechanism of isocitrate dehydrogenase [44]. (**B**) The catalytic mechanism of crotonyl-CoA carboxylase/reductase [45].

Pyruvate synthase (PS, EC 1.2.7.1) and 2-oxoglutarate synthase (OGS, EC 1.2.7.3) are a class of enzymes sharing a similar catalytic mechanism [41,42]. They belong to strictly anaerobic enzymes and show low catalytic activity. Acetyl-CoA can be reductively carboxylated by pyruvate synthase at the expense of two equivalents of ferredoxin to generate pyruvate. Similarly, succinyl-CoA can be converted to 2-oxoglutarate by 2-oxoglutarate synthase [43]. Different from the above two enzymes, isocitrate dehydrogenase (IDH, EC 1.1.1.41/42) converts 2-oxoglutarate to isocitrate at the expense of NAD(P)H [44]. The carboxylation process of isocitrate dehydrogenase is assumed to proceed via the enolate intermediate of 2-oxoglutarate, which is formed with the assistance of divalent metal ions Mg^2+^ or Mn^2+^. After the addition of CO_2_, the unstable keto-tricarboxylic acid intermediate is immediately reduced by NAD(P)H to yield stable isocitrate (Figure 3A). 2-oxoglutarate synthase and isocitrate dehydrogenase are carboxylases of the reductive tricarboxylic acid (rTCA) cycle.

Enoyl-CoA carboxylases/reductases (ECRs) are a class of carboxylases that exist in secondary metabolism, as well as in central carbon metabolism of α-proteobacteria and *Streptomycetes* [45]. The best-studied ECR is crotonyl-CoA carboxylase/reductase (CCR, EC 1.3.1.85) that catalyzes NADPH-dependent reductive carboxylation of crotonyl-CoA into (2S)-ethylmalonyl-CoA. The mechanism of CCR is assumed to proceed via nucleophilic hydride attack at β-carbon of the enoyl-CoA ester; the forming enolate is trapped by CO_2_ to generate (2S)-ethylmalonyl-CoA (Figure 3B). Recently, combining experimental biochemistry, protein crystallography, and advanced computer simulations, Gabriele M. M. Stoffel et al. determined the CO_2_-binding residues at the active site of crotonyl-CoA carboxylase/reductase from *Kitasatospora setae* [46]. Propionyl-CoA synthase from *Erythrobacter* sp. NAP1, as well as an acrylyl-CoA reductase from *Nitrosopumilus maritimus*, have almost no carboxylation activity. Based on the determined CO_2_-binding residues, they used rational design to engineer two enzymes into carboxylases by increasing interactions of the proteins with CO_2_ and suppressing diffusion of water to the active site [47].

**Figure 4:**
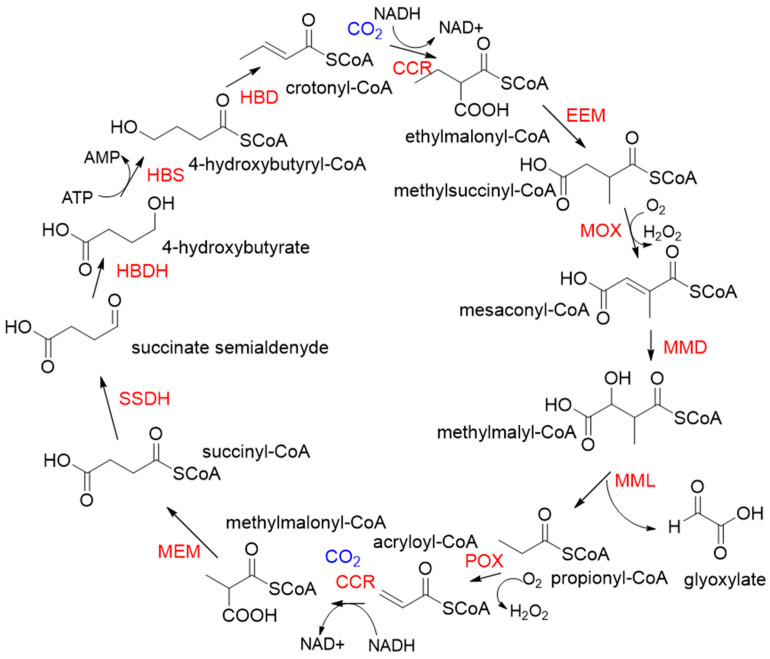
The crotonyl-CoA/ethylmalonyl-CoA/hydroxybutyryl-CoA cycle pathway (CETCH pathway) [21]. CCR, crotonyl-CoA carboxylase/reductase; EEM, ethylmalonyl-CoA epimerase, and mutase; MOX, methylsuccinyl-CoA oxidase; MMD, methylmalyl-CoA dehydratase; MML, methylmalyl-CoA lyase; POX, propionyl-CoA oxidase; MEM, methylmalonyl-CoA epimerase, and mutase; SSDH, succinate semialdehyde dehydrogenase; HBDH, 4-hydroxybutyrate dehydrogenase; HBS, 4-hydroxybutyryl-CoA synthetase; HBD, 4-hydroxybutyryl-CoA dehydratase.

Relative to Rubisco, CCR is oxygen-insensitive, does not react with O_2_, requires only the NADPH, and catalyzes CO_2_ fixation with higher efficiency (K_cat_/K_m_ = 1642.6 s^−1^ mM^−1^) [48,49]. All of these characteristics make CCR a good candidate enzyme for the fixation of CO_2_. Based on CCR, Tobias J. Erb research group constructed a crotonyl-CoA/ethylmalonyl-CoA/hydroxybutyryl-CoA (CETCH) cycle in vitro [21], which consists of 17 enzymes and can convert CO_2_ into organic molecules at a rate of 5 nanomoles of CO_2_ per minute per milligram of protein (Figure 4). Recently, they successfully encapsulated thylakoids isolated from the spinach plant along with all enzymes of the CETCH pathway within water-in-oil droplets [20]. The encapsulated system could use light energy to produce glycolate from CO_2_, while also phosphorylating ADP to ATP.

### 2.4. Substrate-Activated Carboxylases

**Figure 5:**
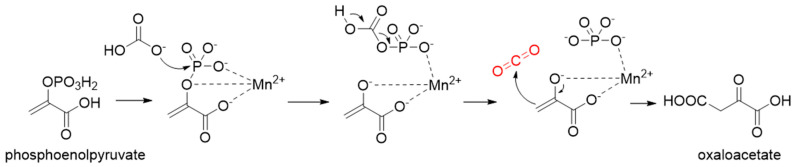
The catalytic mechanism of phosphoenolpyruvate carboxylase [52].

Phosphoenolpyruvate (PEP) carboxylase (PEPC, EC 4.1.1.31) catalyzes the irreversible carboxylation of PEP to form oxaloacetate (OAA) using Mg^2+^ or Mn^2+^ as a cofactor [50]. This kind of enzyme is present in most photosynthetic organisms [51]. PEPC is used to replenish intermediates of the TCA cycle for amino acid biosynthesis, or to shuttle CO_2_ between the mesophyll and bundle sheath cells in C4 plants. The catalytic mechanism of PEPC has been well studied (Figure 5). First, bicarbonate act as a nucleophile to attack phosphate groups in PEP, yielding carboxyphosphate and enolates of pyruvate, which is stabilized by metal ions Mn^2+^. Next, carboxyphosphate decomposes into inorganic phosphate and CO_2_, which is attacked by enolates of pyruvate to form OAA [52].

**Figure 6:**
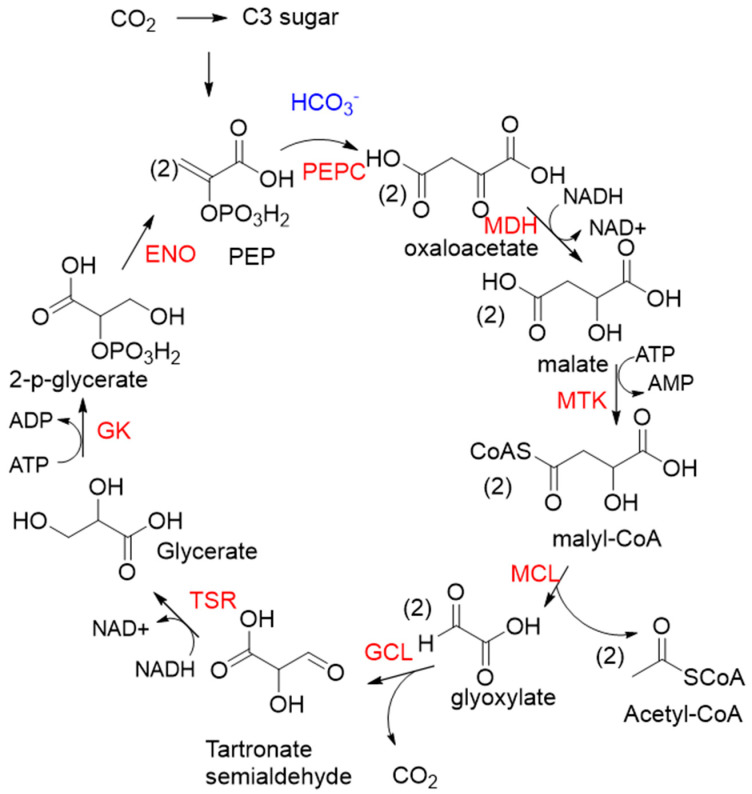
The synthetic malyl-CoA-glycerate (MCG) pathway [19]. PEPC, PEP carboxylase; MDH, malate dehydrogenase; MTK, malate thiokinase; MCL, malyl-CoA lyase; GCL, glyoxylate carboligase; TSR, tartronate semialdehyde reductase; GK, glycerate kinase; ENO, enolase.

PEPC is known to be one of the most active carboxylases (K_cat_/K_m_ = 23,792 s^−1^ mM^−1^) [53]. Based on PEPC, Hong Yu et al. constructed a synthetic malyl-CoA-glycerate (MCG) pathway [19], which is capable of converting one C3 sugar to two acetyl-CoA via fixation of one CO_2_ equivalent, or assimilating glyoxylate, a photorespiration intermediate, to produce acetyl-CoA without carbon loss (Figure 6). Coupling the MCG pathway with the CBB cycle, photosynthetic organisms utilize only 5.5 ATP and 1.5 Rubisco turnovers to produce one acetyl-CoA from CO_2_ equivalents, while the native pathway requires 7 ATP and 3 Rubisco turnovers. When transferring the MCG pathway into a photosynthetic organism *Synechococcus elongates* PCC7942, the intracellular acetyl-CoA level increased, and bicarbonate assimilation was improved by roughly 2-fold.

### 2.5. ThDP-Dependent Carboxylases

**Figure 7:**
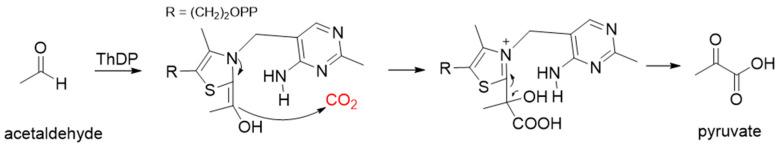
The catalytic mechanism of pyruvate decarboxylase [54,55].

Pyruvate decarboxylase (PDC, EC 4.1.1.1) is a key enzyme of carbon metabolism at the branching point between aerobic respiration and anaerobic alcoholic fermentation, and can be found in some bacteria, yeasts, and plants [54]. PDC catalyzes the decarboxylation of pyruvate by using ThDP and Mg^2+^ as cofactors (Figure 7). This enzyme has been successfully applied to yield pyruvic acid through the reverse carboxylation reaction. To favor the carboxylation, high pH and high bicarbonate concentration are needed [55]. In addition to using a high concentration of bicarbonate solution as a CO_2_ source, elevated CO_2_ pressure is also an effective way to drive the direction of carboxylation. Combining branched-chain α-keto acid decarboxylase (KdcA) from *Lactococcus lactis* with transaminase or amino acid dehydrogenase, Julia Martin et al. achieved the synthesis of L-methionine from the abundant industrial intermediate methional under a 2 bar CO_2_ atmosphere [56].

### 2.6. Multi-Enzyme Complex Constructed Carboxylase

The glycine cleavage system (GCS) is common among many organisms because of its involvement in glycine and serine catabolism [57]. The GCS converts glycine to CO_2_, NH_4_^+^, and methylene-THF. GCS is composed of four proteins, a carrier protein, and three enzymes. They are lipoic acid-containing protein (GcvH), glycine dehydrogenase (GcvP), aminomethyltransferase (GcvT), and lipoamide dehydrogenase (Lpd), respectively. The glycine cleavage process can be divided into three steps. The first step is the decarboxylation of glycine by the glycine dehydrogenase. The decarboxylated moiety is then further degraded by the aminomethyl transferase with the aid of tetrahydrofolate. The last step is the reoxidation of the two sulfhydryl groups to form lipoic acid-generating NADH by dihydrolipomide dehydrogenase. Two sulfhydryl groups or lipoate attached to the lipoic acid-containing protein act as intermediate shuttles.

**Figure 8:**
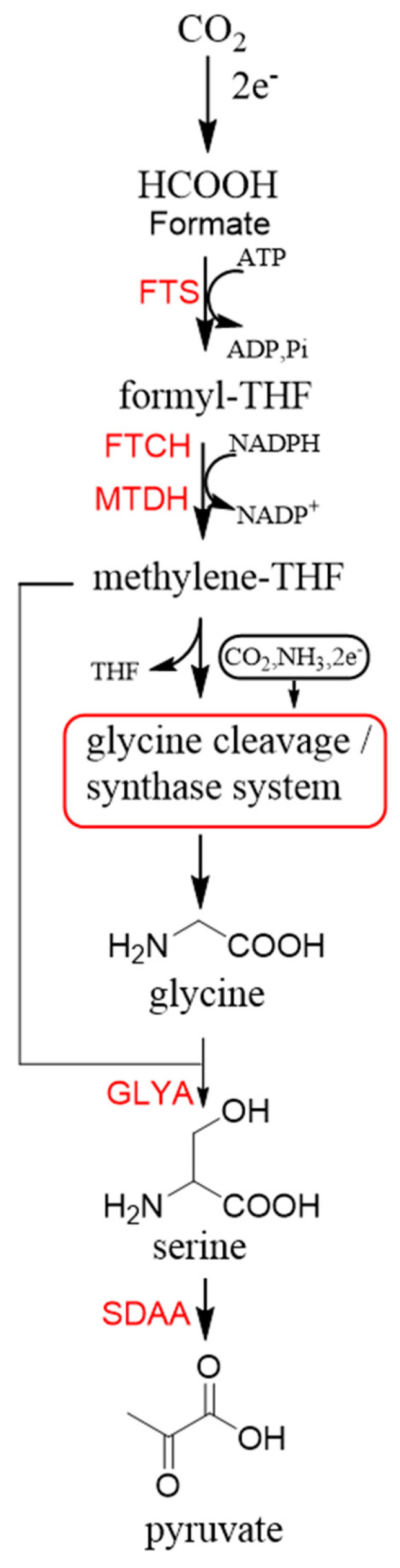
The reductive glycine pathway (RGP) [61]. FTS, formyl-THF synthase; FTCH, formyl-THF cyclohydrolase; MTDH, methylene-THF dehydrogenase; GLYA, L-serine hydroxymethyltransferase; SDAA, L-serine dehydratase.

Now, GCS is confirmed to be reversible (rGCS) and can condense the C1 moiety of methylene-THF with CO_2_ and ammonia to produce glycine [58], which shows great application potential for reductive glycine pathway (RGP). Arren Bar-Even’s research group has made a series of encouraging progress [59,60,61]. Especially, they redesigned the central carbon metabolism of the model bacterium *E. coli* for growth on one-carbon compounds (formate and methanol) using the RGP [61] (Figure 8). Recently, Irene Sánchez-Andrea et al. demonstrated that sulfate-reducing bacterium *Desulfovibrio desulfuricans* (strain G11) could grow autotrophically via the RGP using hydrogen and sulfate as energy substrates [62]. This work first demonstrates that autotrophic microbial growth can be fully supported by RGP, which is a highly ATP-efficient CO_2_ fixation pathway.

### 2.7. prFMN-Dependent Carboxylases

prFMN-dependent decarboxylases catalyze the non-oxidative reversible decarboxylation of aromatic substrates, and play a pivotal role in bacterial ubiquinone (coenzyme Q) biosynthesis and microbial biodegradation of aromatic compounds [63,64]. The prFMN cofactor is provided by an associated prenyltransferase (UbiX), which extends the isoalloxazine FMN ring system through prenylation with a fourth non-aromatic ring. The catalytically active iminium species of the cofactor (prFMN^iminium^) is obtained by oxidizing the reduced prFMN with O_2_. There are two different catalytic reaction mechanisms for the prFMN-assisted (de)carboxylation reaction. For α, β-unsaturated carboxylic acids, the reaction proceeds through the intermolecular 1, 3-dipolar cycloaddition step. While for protocatechuic acid-type substrates, the electrophilic character of the iminium ion of prFMN^iminium^ enables reversible (de)carboxylation via a mono-covalently bound quinoid–cofactor intermediate. prFMN-dependent decarboxylases encompass a wide range of substrates [17], including non-aromatic α, β-unsaturated (acrylic) acid derivatives, catechol, and 4-hydroxybenzoic acid derivatives, polycyclic aromatic hydrocarbons (PAHs), and heterocyclic substrates. Recently, combining ferulic acid decarboxylase (FDC, prFMN-dependent) with carboxylic acid reductase (CAR), alcohol dehydrogenase (ADH), or imine reductase (IRED), Godwin A. Aleku et al. designed cascade reactions to enable efficient functionalization of terminal alkenes to the corresponding aldehyde, alcohol, amide or amine derivatives through ambient CO_2_ fixation [65].

## 3. C-C Ligases for Formaldehyde Biotransformation

**Figure 9:**
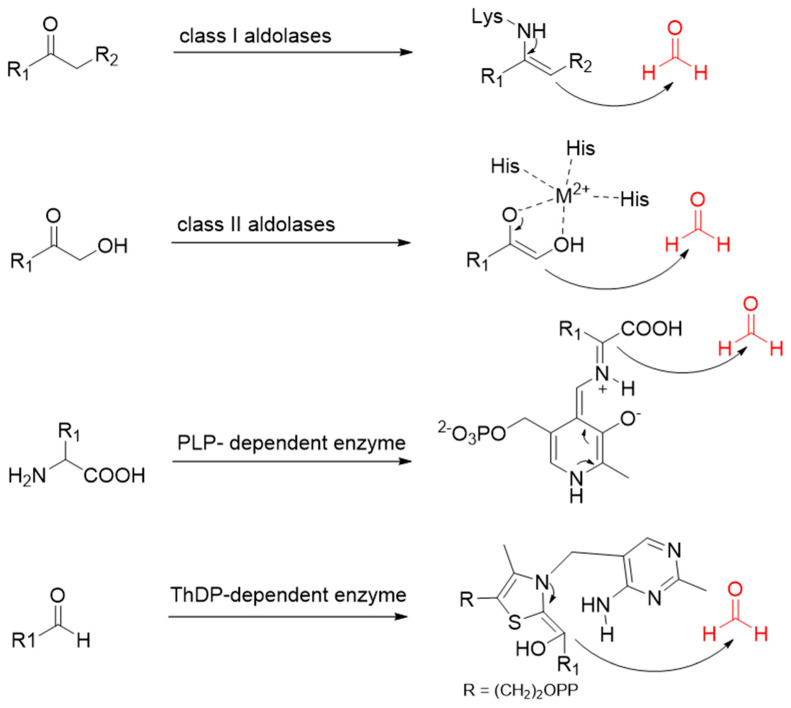
Four types of C-C Ligases for formaldehyde biotransformation [18].

Formaldehyde is a cytotoxic compound and a central metabolic intermediate in methylotrophs [66]. In addition to the naturally occurring formaldehyde condensing enzymes, such as dihydroxyacetone synthase (DAS), 3-hexulose-6-phosphate synthase (HPS), and serine hydroxymethyltransferase (SHMT), several promiscuous enzymes display catalytic activity towards formaldehyde. Based on the catalytic mechanism, those enzymes can be divided into 4 categories (Figure 9). Class I aldolases activate the donor substrate with a lysine residue. Class II aldolases use a metal cofactor to facilitate the activation of donor substrate to an enolate. The third class is hydroxymethyl pyridoxal 5′-phosphate (PLP) dependent enzymes. The PLP coenzyme activates the donor substrate to form a quinoide-aldimine intermediate. The last is ThDP-dependent enzymes. The ThDP coenzyme activates the donor substrate to form an enamine/carbanion intermediate. For the first three classes of enzymes, formaldehyde cannot act as a donor substrate because it is not enolizable. For ThDP-dependent enzymes, formaldehyde can act either as a donor or an acceptor. C-C ligases using formaldehyde as a donor or acceptor were summarized in Table 2. Furthermore, the formaldehyde assimilation pathways published in recent years are highlighted.

### 3.1. Class I Aldolases for Formaldehyde Biotransformation

Natural class I aldolases are generally promiscuous. It has been confirmed that several class I aldolases can tolerate formaldehyde as an acceptor substrate. From the perspective of biocatalysis, the promiscuity of class I aldolases provides solutions for producing valuable chemicals from formaldehyde. The following are examples of class I aldolases catalyzing formaldehyde as a receptor. 4-hydroxy-2-oxoglutarate aldolase (EC 4.1.3.16, class I aldolase) can catalyze the aldol addition of pyruvic acid or phenylpyruvic acid to formaldehyde [18]. DHAP-dependent aldolases, including D-Fructose-1, 6-bisphosphate (FBP) aldolases (FruA, EC 4.1.2.13), and tagatose 1, 6-diphosphate aldolase (TagA, EC 4.1.2.40), can catalyze the aldol addition of dihydroxyacetone phosphate (DHAP) to formaldehyde [67,68]. D-Fructose-6-phosphate aldolase (FSA, EC 4.1.2.n) can catalyze the aldol addition of dihydroxyacetone (DHA), hydroxyacetone (HA), and glycolaldehyde (GA) to formaldehyde [69].

### 3.2. Class II Aldolases for Formaldehyde Biotransformation

**Figure 10:**
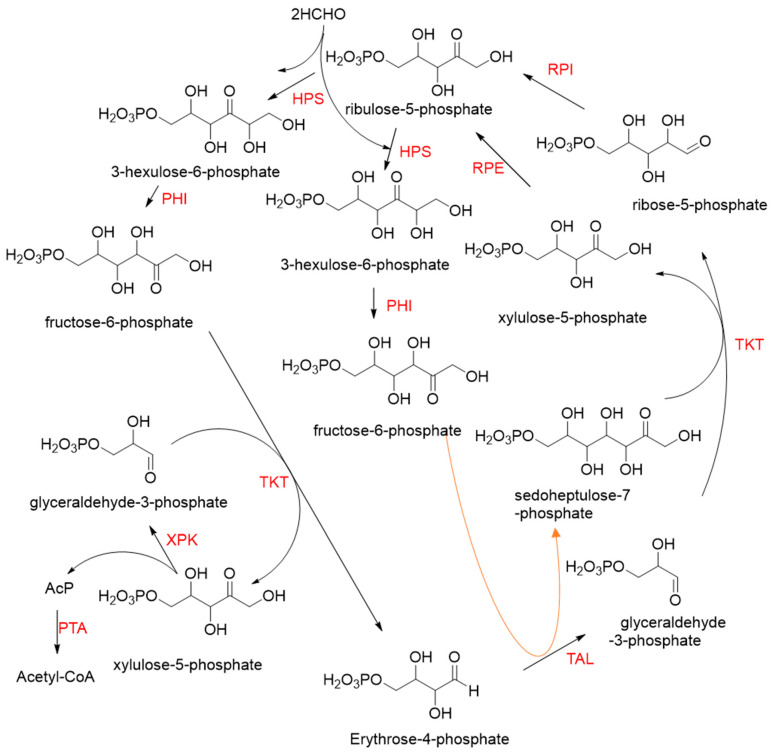
The methanol condensation cycle (MCC) [70]. HPS, hexulose phosphate synthase; PHI, phosphohexulose isomerase; TAL, transaldolase; TKT, transketolase; RPE, D-ribulose 5-phosphate epimerase; RPI, Ribose-5-phosphate isomerase; XPK, phosphoketolase; PTA, phosphate acetyltransferase.

Hexulose phosphate synthase (HPS, EC 4.1.2.43) is a class II aldolase found in aerobic methylotrophic bacteria and is involved in the ribulose monophosphate (RuMP) cycle. It catalyzes D-ribulose 5-phosphate with formaldehyde to yield D-arabinose 3-hexulose 6-phosphate. Due to strict substrate specificity, it is difficult to use hexulose phosphate synthase to synthesize value-added chiral products. While, by combining the non-oxidative glycolysis (NOG) with the RuMP, Igor W. Bogorad et al. constructed a methanol condensation cycle (MCC) [70], which can convert methanol to higher-chain alcohols or other acetyl-CoA derivatives using enzymatic reactions in a carbon-conserved and ATP-independent system (Figure 10). It is generally believed that the RuMP cycle is the most efficient naturally occurring route for methanol assimilation. However, realizing the heterogeneous construction of the RuMP cycle is a challenging task. Recently, a series of progress has been made [71,72]. By using metabolic robustness criteria followed by laboratory evolution, Frederic Y.-H. Chen et al. enabled the growth of the engineered *E. coli* with methanol as the sole carbon source [72].

**Figure 11:**
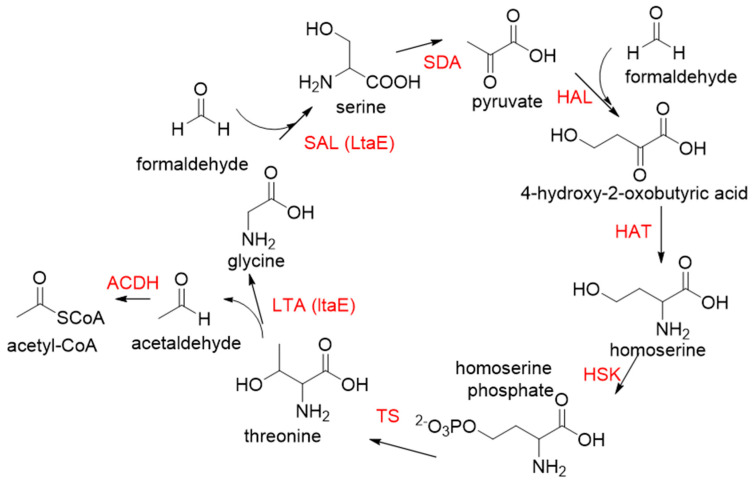
The homoserine cycle [74]. SAL, serine aldolase; SDA, serine deaminase; HAL, 4-hydroxy-2-oxobutanoate (HOB) aldolase; HAT, HOB aminotransferase; HSK, homoserine kinase; TS, threonine synthase; LTA, threonine aldolase; ACDH, acetylating acetaldehyde dehydrogenase. Both SAL and LTA are catalyzed by the same LtaE enzyme.

Another class II aldolase, 2-keto-3-deoxy-L-rhamnonate aldolase (YfaU, EC 4.1.2.53), can also catalyze pyruvate with formaldehyde to generate 4-hydroxy-2-oxobutanoate. Combining YfaU with (S)-or (R)-selective transaminases, Karel Hernandez et al. achieved the stereoselective synthesis of (S)-and (R)-homoserine with high yield from formaldehyde and alanine [73]. Relying on the aldol reaction of pyruvate with formaldehyde as a superior synthetic pathway design, Hai He et al. constructed the homoserine cycle [74], which can assimilate two molecules of formaldehyde or methanol to generate one molecule of acetyl-CoA (Figure 11). Compared to the RuMP cycle, the homoserine cycle support higher theoretical yields of products that are derived from acetyl-CoA, including ethanol, acetone, butyrate, butanol, citrate, itaconate, 2-ketoglutarate, and levulinic acid.

### 3.3. PLP-Dependent Aldolases for Formaldehyde Biotransformation

Serine hydroxymethyltransferase (SHMT, EC 2.1.2.1) belongs to the PLP-dependent aldolase family and is involved in serine cycle and reductive glycine pathway (RGP) [75,76]. The natural serine cycle pathway found in *Methylobacterium extorquens AM1* can assimilate one molecule of formaldehyde or methanol and one molecule of bicarbonate into acetyl-CoA. Hong Yu et al. constructed a modified serine cycle [77], which uses formaldehyde dehydrogenase (Faldh) to simplify the oxidation of formaldehyde to formate, and also utilize the combination of alanine-glyoxylate transaminase and serine dehydratase to avoid hydroxypyruvate as an intermediate in the conversion from glyoxylate to PEP. By utilizing the modified serine cycle, they achieved the conversion of methanol to ethanol in an engineered *E. coli* strain.

Similar to SHMT, there are two types of PLP-dependent enzymes that can achieve the synthesis of unnatural amino acids. α-methylserine hydroxymethyltransferase (MSHMT, EC 2.1.2.7) catalyzes the formation of α-methyl-L-serine from D-alanine and formaldehyde [78]. α-Methylserine aldolase can achieve the enantioselective formation of α-methyl-L-serine and α-ethyl-L-serine from D-alanine and D-butanine with formaldehyde [79]. Different from SHMT and MSHMT, α-Methylserine aldolase is tetrahydrofolate (THF)-independent in vitro.

### 3.4. ThDP-Dependent C-C Ligases for Formaldehyde Biotransformation

Dihydroxyacetone synthase (DAS, EC 2.2.1.3) belongs to the ThDP-dependent enzymes and is involved in xylulose monophosphate pathway (XuMP). It catalyzes D-xylulose 5-phosphate with formaldehyde to yield D-glyceraldehyde 3-phosphate and dihydroxyacetone [80]. The catalytic process can be described as follows: The activated ThDP (C2 of the ThDP is deprotonated) attack the carbonyl group of D-xylulose 5-phosphate, and then the bond between C-2 and C-3 of D-xylulose 5-phosphate break with the assistance of basic residues, generating 2-α, β-dihydroxyethylidene-THDP (DHETHDP, enamine or carbanion intermediate). DHETHDP reacts with formaldehyde to gain dihydroxyacetone. Currently, DAS is not exploited as a biocatalyst. However, *Pichia pastoris*, a kind of methylotrophic yeast, have been engineered to produce chemicals from methanol using XuMP [81]. Similar to DAS, transketolase (TK, EC 2.2.1.1) can catalyze the reaction of D-fructose-6-phosphate or L-sorbose with formaldehyde to generate dihydroxyacetone [82].

**Figure 12:**
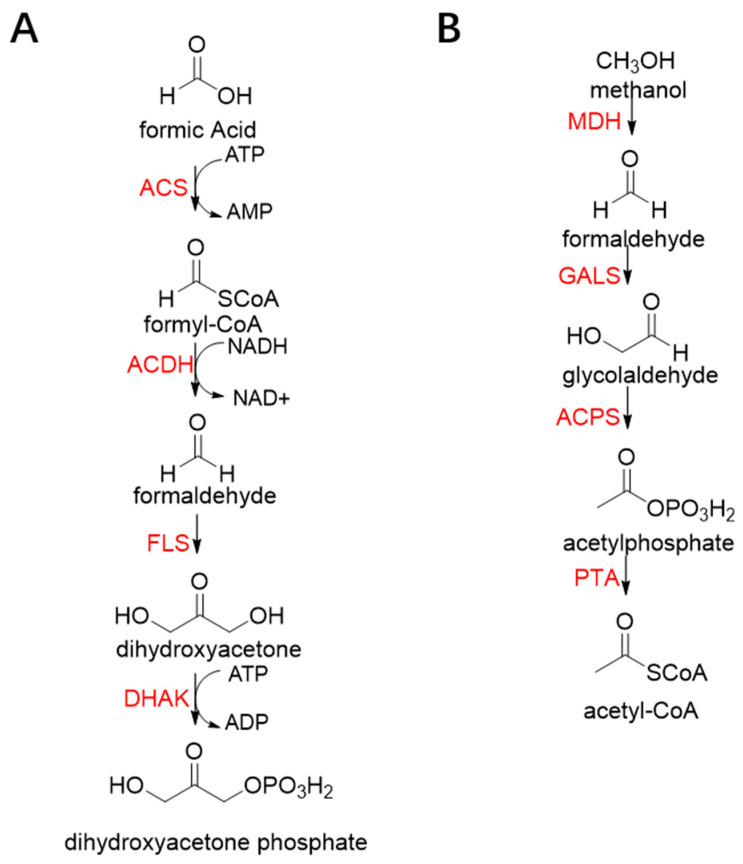
The formolase (FLS) pathway [22] and synthetic acetyl-CoA (SACA) pathway [23]. (**A**) The formolase (FLS) pathway. ACS, acetyl-CoA synthase; ACDH, acetaldehyde dehydrogenase; FLS, formolase; DHAK, dihydroxyacetone kinase. (**B**) The synthetic acetyl-CoA (SACA) pathway. GALS, glycolaldehyde synthase; ACPS, acetyl-phosphate synthase; PTA, phosphate acetyltransferase.

The benzaldehyde lyase (BAL, EC 4.1.2.38) catalyzes the reversible ligation of two benzaldehyde to yield an (R)-benzoin [83]. When formaldehyde acts as a receptor, benzaldehyde first reacts with formaldehyde. However, wild-type BAL was shown to catalyze the oligomerization of formaldehyde into glycolaldehyde and dihydroxyacetone with low activity. Justin B. Siegel et al. engineered the BAL through computationally design and gained formolase (FLS) with high formaldehyde catalytic activity [22]. By combining FLS with acetyl-CoA synthase (ACS), acetaldehyde dehydrogenase (ACDH), and dihydroxyacetone kinase (DHAK), they created an FLS pathway, which assimilates formate into dihydroxyacetone phosphate with four steps (Figure 12A). Compared with the natural carbon fixation pathway, the FLS pathway has obvious advantages in chemical driving force, biomass yield, and reaction conditions (aerobic).

Benzoylformate decarboxylase (BFD, EC 4.1.1.7) is also a ThDP-dependent enzyme that catalyzes the conversion of benzoylformate to benzaldehyde and CO_2_ [84]. When formaldehyde is the only substrate, trace amounts of glycolaldehyde can be detected. Our research group engineered the BFD through directed evolution and gained glycolaldehyde synthase (GALS) with high catalytic activity for formaldehyde [23]. Different from FLS, GALS enables synthesis of glycolaldehyde even under high formaldehyde concentration conditions. By combining GALS with repurposed phosphoketolase (ACPS), and phosphate acetyltransferase (PTA), we constructed a synthetic acetyl-CoA (SACA) pathway, which assimilates formaldehyde into acetyl-CoA with only three steps (Figure 12B). Compared to the FLS pathway, SACA has obvious advantages in the synthesis of acetyl-CoA derivatives. When dihydroxyacetone phosphate (C3), the product of the FLS pathway, is converted to acetyl-CoA, one carbon is lost as CO_2_. Although both the FLS pathway and SACA pathway have shown great potential in assimilating C1 compounds, the low activity and affinity of FLS and GALS towards formaldehyde restrict their further application in vivo. Recently, Alexander Chou et al. reported that 2-hydroxyacyl CoA lyase (HACL) could catalyze the ligation of formyl-CoA with formaldehyde to produce glycolyl-CoA [85]. Compared to FLS and GALS, HACL shows higher catalytic activity and affinity towards formaldehyde. Although they mainly focus on the bioconversion of formaldehyde to glycolate, perhaps this alternative pathway will be available in vivo in the future.

## 4. C-C Ligases for CO and Formate Biotransformation

CO and formate are relatively inert compounds, and few enzymes can catalyze the C-C forming with them. However, they are ideal electron donors [86], which can be easily oxidized to CO_2_ and reduce power. The reducing power can support CO_2_ fixation and serves to provide the cell with energy. Several microbes can grow on formate or CO as the sole carbon source based on this strategy. CO is a toxic and flammable gas with low solubility, while formic acid is readily soluble and of low toxicity. So formic acid is a preferred carbon source and mediator of electrons.

### 4.1. C-C Ligases for CO Biotransformation

Diverse microbes can grow on CO as the sole carbon source, including anaerobes, such as *Moorella thermoacetica*, some purple sulfur bacteria akin to *Rhodospirillum rubrum*, and *Carboxydothermus hydrogenoformans*, as well as some aerobic carboxydobacteria like *Oligotropha carboxidovorans* [87]. For aerobes, CO is first oxidized to CO_2_ by Mo-Cu−CODH (CO dehydrogenase), and then CO_2_ is fixed by the CBB cycle. While for anaerobic microbes, CO is oxidized to CO_2_ by Ni−CODH, and the CO_2_ is then fixed by the WL pathway. Only CO dehydrogenase/acetyl-CoA-synthase complex existing in WL pathway can directly achieve the C-C extension [88]. CO dehydrogenase (CODH) reversibly catalyzes CO_2_ reduction into CO, and then acetyl-CoA synthase (ACS) catalyzes the condensation of in situ generated CO with CoA and a methyl group bound to the cobalt center in a B_12_-containing protein to generate acetyl-CoA. The WL pathway is not only a predominant CO_2_ sink under anaerobic conditions, but also used to synthesize desired products, such as ethanol and 2, 3-butanediol from industrial waste gases (mainly CO, CO_2_, H_2_) [89]. In addition to chemical energy, several anaerobic bacteria, such as *Sporomusa ovata*, and *Moorella thermoacetica*, can employ light energy to enable the photosynthesis of acetic acid from CO_2_ [90,91,92].

### 4.2. C-C Ligases for Formate Biotransformation

Similar to CO assimilation, the CBB cycle and WL pathways are two carbon-fixation pathways known to support formatotrophic growth (i.e., growth on formate) by full oxidation of formate [93]. Different from CO, formate can not only be oxidized to CO_2_ to achieve carbon fixation, but also can be reduced to methylene-THF in vivo. The serine pathway in methylotrophic organisms is also known to support formatotrophic growth [94,95]. The metheylene-THF can spontaneously release formaldehyde, so RuMP, XuMP, RGP, FLS, and SACA pathways are, thus, highly promising for supporting formatotrophic growth. Formate is a relatively strong acid (pKa = 3.75), and normally it is deprotonated. So formate is a much poorer electrophile than CO_2_ [88]. The direct formate-fixing reactions are rare. Only one formate-fixing reaction catalyzed by pyruvate formate-lyase (PFL) is confirmed. This enzyme is mostly known to support pyruvate cleavage, producing an extra ATP molecule during anaerobic sugar fermentation. Now the reversibility of PFL has been demonstrated. Especially, the enzyme was shown to achieve the condensation of acetyl-CoA and formate in vivo [96], supporting efficient growth of *E. coli* on acetate and formate. The catalytic mechanism of PFL is a radical process, in which a single electron is extracted from formate by cysteine radical. The resulting formyl radical then attacks an enzyme-bound acetate moiety to generate a pyruvyl radical, which is released as pyruvate.

## 5. Conclusions

Newly mined or designed carboxylases and C-C ligases not only greatly expand the scope of C1 transformation in vitro, but also are expected to improve the efficiency of C1 assimilation in vivo. For example, mined prFMN-dependent (de)carboxylases enable efficient functionalization of terminal alkenes to the corresponding aldehyde, alcohol, amide, or amine derivatives through ambient CO_2_ fixation [65]. The reversible glycine cleavage system (GCS) enables the growth of *E. coli* on one-carbon compounds (formate and methanol) using the reductive glycine pathway, which is theoretically the most efficient route for formate assimilation to date [61]. With the development of synthetic biology, we believe that more and more carboxylases and C-C ligases are being mined or designed.

Compared to C1 transformation in vitro, constructing an efficient C1 assimilation pathway is more desirable. Since an efficient C1 assimilation pathway will be possible to greatly reduce industrial production cost and also alleviate the pressure of resource supplement for bio-manufacturing in the future. It is encouraging that the constructed CETCH pathway based on crotonyl-CoA carboxylase/reductase, can use light energy to produce multi-carbon molecule glycolate from CO_2_, while also phosphorylating ADP to ATP. Although several artificial pathways, such as the homoserine cycle, formolase pathway (FLS), and synthetic acetyl-CoA (SACA) pathway, show great application potential, model strains (*E. coli*) that integrate these artificial pathways have low growth efficiency when C1 compounds is used as carbon source or energy source. Of course, a lot of work needs to be done to make these artificial pathways truly applied to industrial C1 biotransformation.

C1 compounds (CO_2_, HCOOH, HCHO, CH_3_OH, and CH_4_) can be interconverted. In methanotrophs, CH_4_ can be continuously oxidized to CO_2_ and then assimilated through the CBB cycle. In methanogens, CO_2_ can be continuously reduced to CH_4_ [97]. CO_2_ also can be continuously reduced to CH_3_OH in vitro [98]. Therefore, constructing a new assimilation pathway based on any of the C1 compounds can theoretically be used to assimilate other C1 compounds. However, among C1 compounds, only CO_2_ and formaldehyde are good candidate substrates for exploiting carboxylases and C-C ligases. For the CO_2_ assimilation pathway, the energy source is an issue that must be considered. Both electric energy and light energy may be ideal energy sources. Recently, the semiconductor–bacteria biohybrid photosynthetic system was reported to efficiently realize the synthesis of acetic acid from CO_2_ with the non-photosynthetic bacteria [90]. Formaldehyde exhibits high and versatile reactivity, in comparison to other C1 compounds. However, formaldehyde is toxic to the cell, therefore, the mined or designed C-C ligases must have high activity and affinity towards formaldehyde.

## Figures and Tables

**Table 1:** Representative carboxylases for CO_2_ fixation.

Aliphatic Substrates	Product	Enzyme and Category	Pathway
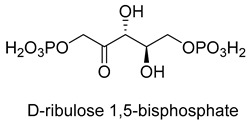	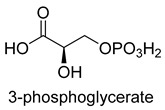	Rubisco EC 4.1.1.39 Only divalent metal-dependent carboxylase	Calvin–Benson–Bassham (CBB) cycle
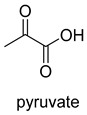	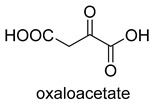	PC EC 6.4.1.1 ATP-dependent carboxylase	/
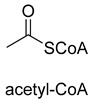	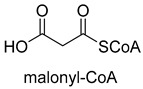	ACC EC 6.4.1.2 ATP-dependent carboxylase	3-hydroxypropionate cycle (HP) and 3-HP/4-hydroxybutyrate cycle (HB)
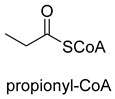	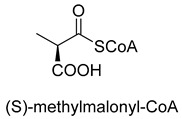	PCC EC 6.4.1.3 ATP-dependent carboxylase	3-HP and 3-HP/4-HB
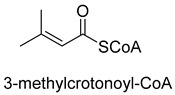	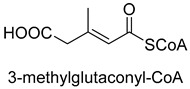	MCC EC 6.4.1.4 ATP-dependent carboxylase	/
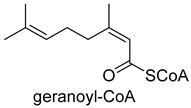	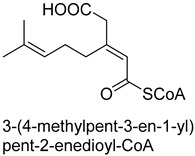	GCC EC 6.4.1.5 ATP-dependent carboxylase	/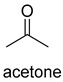	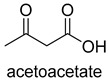	AC EC 6.4.1.6 ATP-dependent carboxylase	/
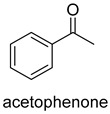	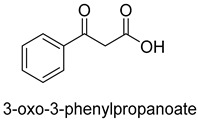	APC EC 6.4.1.8 ATP-dependent carboxylase	/
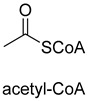	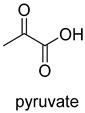	PS EC 1.2.7.1 Redox equivalents-dependent carboxylases	4-HB
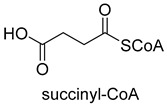	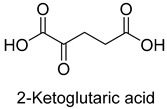	OGS EC 1.2.7.3 Redox equivalents-dependent carboxylase	rTCA
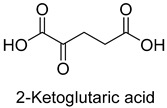	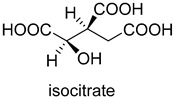	IDH EC 1.1.1.41/42 Redox equivalents-dependent carboxylase	rTCA
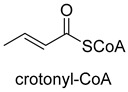	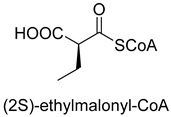	CCR EC 1.3.1.85 Redox equivalents-dependent carboxylase	CETCH pathway
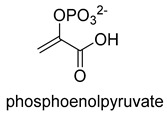	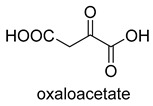	PEPC EC 4.1.1.31 Substrate-activated carboxylase	4-HB and MCG pathway
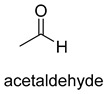	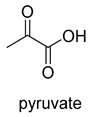	PDC EC 4.1.1.1 ThDP-dependent carboxylase	/
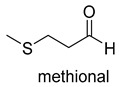	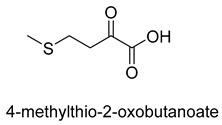	KdcA from *Lactococcus lactis* ThDP-dependent carboxylase	/
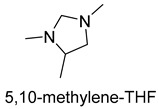	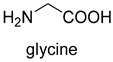	GCS Multi-enzyme complex constructed carboxylase	reductive glycine pathway
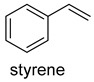	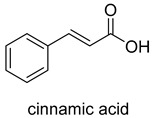	Ferulic acid decarboxylase (FDC1) prFMN-dependent carboxylase	/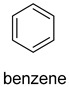	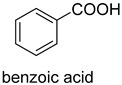	Benzene carboxylase prFMN-dependent carboxylase	/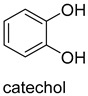	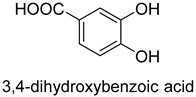	3,4-dihydroxybenzoic acid decarboxylase from E. cloacae (EcAroY) prFMN-dependent carboxylase	/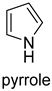	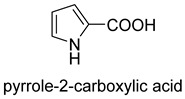	Pyrrole-2-carboxylic acid decarboxylase prFMN-dependent carboxylase	/

**Table 2:** Summary of C-C Ligases with formaldehyde as a donor or acceptor.

Substrates	Product	Enzyme and Category	Pathway
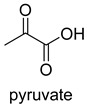	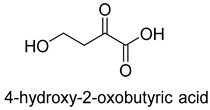	4-hydroxy-2-oxoglutarate aldolase (EC 4.1.3.16) class I aldolase	/
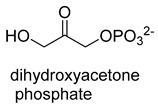	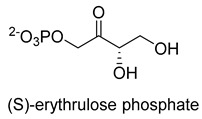	D-Fructose-1,6-bisphosphate (FBP) aldolases (FruA, EC 4.1.2.13), and Tagatose 1,6-diphosphate aldolase (Tag A, EC 4.1.2.40): class I aldolase	/
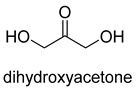	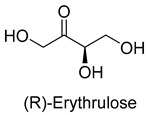	D-Fructose-6-phosphate aldolase (FSA) class I aldolase	/
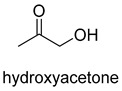	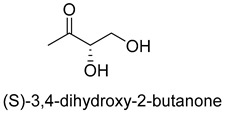	D-Fructose-6-phosphate aldolase (FSA) class I aldolase	/
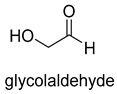	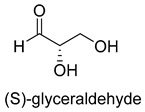	D-Fructose-6-phosphate aldolase (FSA) class I aldolase	/
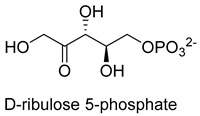	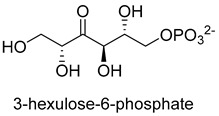	Hexulose phosphate synthase (HPS, EC 4.1.2.43) class II aldolase	RuMP
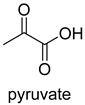	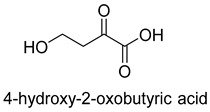	2-keto-3-deoxy-L-rhamnonate aldolase (YfaU, EC 4.1.2.53) class II aldolase	the homoserine cycle
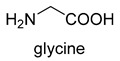	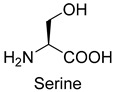	Serine hydroxymethyltransferase (SHMT, EC 2.1.2.1) PLP-dependent aldolase	serine cycle and RGP
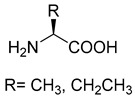	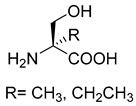	MSHMT for D-alanine. α-Methylserine aldolase for D-alanine and D-butanine PLP-dependent aldolase	/
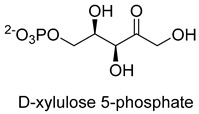	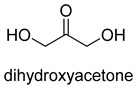	Dihydroxyacetone synthase (DAS, EC 2.2.1.3) ThDP-dependent C-C Ligase	XuMP
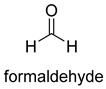	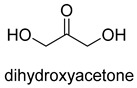	Formolase (FLS) ThDP-dependent C-C Ligase	FLS pathway
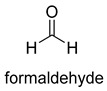	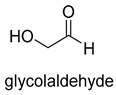	Glycolaldehyde synthase (GALS) ThDP-dependent C-C Ligase	SACA pathway
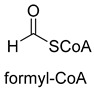	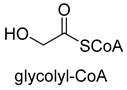	2-hydroxyacyl CoA lyase (HACL) ThDP-dependent C-C Ligase	/
